# Effect of Minimum Bronchial Cuff Volume of Left-Sided Double-Lumen Tube for One-Lung Ventilation on the Change in Bronchial Cuff Pressure during Lateral Positioning in Thoracic Surgery: A Prospective Observational Study

**DOI:** 10.3390/jcm12072473

**Published:** 2023-03-24

**Authors:** Young-Woo Do, Jong-Hae Kim, Kyungmin Kim, Jinyoung Oh, Kyung-Hwa Kwak, Younghoon Jeon, Sung-Hye Byun

**Affiliations:** 1 Department of Thoracic and Cardiovascular Surgery, School of Medicine, Kyungpook National University, 807, Hoguk-ro, Buk-gu, Daegu 41404, Republic of Korea; 2 Department of Anesthesiology and Pain Medicine, School of Medicine, Daegu Catholic University, 33, Duryugongwon-ro 17-gil, Nam-gu, Daegu 42472, Republic of Korea; 3 Department of Anesthesiology and Pain Medicine, School of Medicine, Kyungpook National University, 807, Hoguk-ro, Buk-gu, Daegu 41404, Republic of Korea; 4 Department of Anesthesiology and Pain Medicine, School of Dentistry, Kyungpook National University, 130, Dongdeok-ro, Jung-gu, Daegu 41944, Republic of Korea

**Keywords:** double-lumen tube, lateral position, cuff, pressure, thoracic surgery

## Abstract

The minimum bronchial cuff volume (BCVmin) of a double-lumen tube (DLT) without air leaks during lung isolation may vary among individuals, and lateral positioning could increase the bronchial cuff pressure (BCP). We investigated the effect of initially established BCVmin (BCVi) on the change in BCP by lateral positioning. Seventy patients who underwent elective lung surgery were recruited and divided into two groups according to the BCVi obtained during anesthetic induction in each patient. Outcome analysis was conducted using data from 39 patients with a BCVi greater than 0 (BCVi > 0 group) and 27 with a BCVi of 0 (BCVi = 0 group). The primary outcome was a change in the value measured in the supine and lateral positions of the initially established BCP (BCPi; BCP at the time of BCVi injection), which was significantly larger in the BCVi > 0 group than in the BCVi = 0 group (1.5 (0.5–6.0) cmH_2_O vs. 0.0 (0.0–1.0) cmH_2_O; *p* < 0.001). BCVi was related to the left main bronchus (LMB) diameter (Spearman’s rho = 0.676, *p <* 0.001) and the gap between the LMB diameter and the outer diameter of the bronchial cuff (Spearman’s rho = 0.553, *p* < 0.001). Therefore, selecting a DLT size with a bronchial cuff that fits each patient’s LMB may be useful in minimizing the change in BCP when performing lateral positioning during thoracic surgery. If the bronchial cuff requires unavoidable initial inflation, it is necessary to be aware that BCP may increase during lateral positioning and to monitor the BCP regularly if possible.

## 1. Introduction

Overinflation of the bronchial cuff of the double-lumen tube (DLT) can cause damage to the airway mucosa and a misplacement of the tube [1,2,3], and underinflation may cause an incomplete collapse of the non-ventilated lung and incomplete ventilation of the lung requiring ventilation [4]. The appropriate cuff pressure is generally in the range of 20–30 cmH_2_O [1,5]; however, in a study by Okubo et al., who observed that the minimum value of bronchial cuff volume (BCV) and pressure using capnography, the lung isolation was deemed possible at pressure lower than 25 cmH_2_O (the generally recommended tube cuff pressure) in both men and women [6]. When checking for air leakage using the capnography waveform, in a recent study by Yamada et al., the BCV satisfying airtight seal was significantly smaller than that of a pressure-guided method for inflating the bronchial cuff with 20 cmH_2_O [7].

The minimum BCV (BCVmin) at which such air leakage does not occur may vary among individuals. This is presumably because the diameter of the left main bronchus (LMB) varies among individuals, which results in variation in the gap between the diameter of the LMB and the outer diameter of the mounted DLT. Moreover, our previous study revealed that lateral positioning could increase the pressure of the bronchial cuff mounted on the LMB owing to gravity-induced morphological and conformational changes in the trachea [2].

Considering these factors, we hypothesized that the changes in the bronchial cuff pressure (BCP) owing to a positional change might depend on the initially established BCVmin (BCVi) in the supine position. Therefore, we investigated the effect of BCVi on the change of BCP due to the positional change from the supine to the lateral decubitus position by dividing patients into two groups based on whether the BCVi was 0 or greater than 0.

## 2. Materials and Methods

This prospective, single-center observational study was approved by the Institutional Review Board of Kyungpook National University Chilgok Hospital (IRB No: KNUCH 2021-08-059-001) on 8 October 2021. The study protocol was registered at www.clinicaltrial.gov (NCT05222568). This study was conducted at a tertiary university hospital in Daegu, South Korea, from October 2021 to December 2021.

### 2.1. Participants

Patients aged 18–80 years, with an American Society of Anesthesiologists (ASA) physical status of 1–3 and scheduled to undergo elective lung surgery requiring lateral decubitus positioning and one-lung ventilation (OLV) using a left-sided DLT, were enrolled. Written informed consent was obtained from each participant or a legally authorized representative one day prior to surgery.

Patients who required a right-sided DLT and those with an intraluminal lesion in the LMB, an anatomical obstruction in the tracheobronchial tree, impaired lung compliance, severe respiratory dysfunction, or body mass index (BMI) ≥ 30 kg/m^2^ were excluded.

This is a prospective observational study. The patients were divided into two groups with BCVi = 0 or BCVi > 0, which was the BCVi naturally reached during anesthetic induction in each patient and not intentionally set by the researchers.

### 2.2. Preoperative Computed Tomography (CT) Analysis

For the internal diameter of the LMB, the vertical distance between the parallel anterior and posterior walls of the LMB at 2–5 slices under the carina was measured using preoperative chest computed tomography (CT) on an image magnified by 10 times. Since the size and length of the tissue can be expressed differently based on the window setting where the CT image is displayed [8,9,10], each measurement was performed at the lung and mediastinal window and their average value was obtained. This process was performed by two investigators independent of the investigators who measured intraoperative outcomes, and the average value was calculated as the LMB diameter. In this study, a DLT of 37 and 35 Fr for males and females, respectively, was employed. After measuring the internal diameter of the LMB of each patient, the gap between the LMB and the DLT was calculated by subtracting the outer diameter (10.1 mm for 37 Fr and 9.6 mm for 35 Fr [11]) of the tube used for that patient.

### 2.3. Anesthesia and DLT Intubation

On the day of surgery, all patients were equipped with standard monitoring devices, such as electrocardiograms, pulse oximeters, and non-invasive blood pressure measurement apparatuses. Total intravenous anesthesia using propofol and remifentanil with target-controlled infusion was performed. During anesthesia, the effective-site concentrations of propofol and remifentanil were adjusted at the discretion of the anesthesiologist according to the bispectral index level and hemodynamic status of the patient. After administering 0.8 mg/kg of rocuronium for muscle relaxation, tracheal intubation was performed using a left-sided DLT (Broncho-Cath^®^, Mallinckrodt Medical Ltd., Athlone, Ireland). The size of the DLT was 37 and 35 Fr for males and females, respectively. The proper placement of the DLT was confirmed by two experienced investigators (J.Y.O., S.H.B.) with at least 5 years of experience in thoracic anesthesia using a 4.0-mm fiberoptic bronchoscope (FOB; Karl Storz Endoskope, Tuttlingen, Germany) according to the method described by Campos et al. [12]; thereafter, the tube was temporarily fixed at the patient’s mouth using tape while inflating the cuff of the tube.

### 2.4. Outcome Measurements

The pressure of the bronchial cuff of the DLT was measured using a cuff manometer (VBM Medizintechnik GmbH™, Sulz, Germany), which was assembled to connect to the valve of the pilot balloon of the bronchial cuff via a three-way stopcock to inflate air into the cuff. Each measurement was performed for 2 min following the confirmation of DLT placement in each posture, including supine and lateral decubitus positions. Before air injection into the cuff, the intracuff pressure was equilibrated at atmospheric pressure to avoid generating a negative pressure in the cuff. The BCP was assessed while inflating the cuff with air in 0.5 mL increments, starting at 0 mL, until the BCV without air leak was found under the OLV condition by clamping the lumen of the operative side of the DLT. If the pressure was expressed as a range, the average of the maximum and minimum values was considered the BCP. Whether air leaked around the bronchial cuff was also checked at each time point when inflating the cuff in 0.5 mL increments. To detect air leaks, simple methods, including capnography, evaluating the pressure–volume loop on the respiratory monitor of the anesthetic machine, or measurement of the exhaled return tidal volume (TVe), were employed, as described in a previous study [2]. If the configuration on capnography or the pressure–volume loop was distorted or TVe was delivered at less than 80% of the established tidal volume, an air leak was deemed to be present. The smallest BCV without an air leak and the pressure at that timepoint were denoted as BCVmin and BCPmin, respectively. In the present study, BCVmin measured in the supine position was termed BCVi, and the pressure when injecting the BCVi at any timepoint was termed the BCPi. Following the measurement of these values, the bronchial cuff was aspirated completely and equilibrated at atmospheric pressure, thereby returning to its resting state under two-lung ventilation. Ventilation was maintained at a tidal volume of 6–8 mL/kg, and the respiration rate was adjusted to maintain an end-tidal carbon dioxide value of 30–35 mmHg; a positive end-expiratory pressure (PEEP) of 5 cmH_2_O was also applied. The ventilatory settings were constantly maintained in both patient positions during OLV to control the variables that could affect cuff pressure, such as peak inspiratory pressure (PIP).

After lateral positioning of the patient with an axillary roll placed under the dependent axilla, the operating table was flexed enough to maximally widen the intercostal space. All the positioning procedures were performed by an independent senior resident. The bronchial cuff was placed at the same location using the FOB by the same investigator before and after the lateral positioning of the patient. The BCP and air leak were checked while inflating the cuff in the same manner as in the supine position, and following the assessment of the BCVmin, BCPmin, and BCPi, the cuff manometer remained connected to the pilot balloon until the start of the operation. Considering the tracheal cuff of the DLT, the cuff pressure was adjusted to a range of 20–30 cmH_2_O using a cuff manometer whenever the tracheal cuff was inflated after confirming the DLT location in the lateral decubitus position in the present study since our previous study already confirmed a change in the tracheal cuff pressure following lateral positioning [2].

Two minutes after the incision and insertion of the trocar for thoracoscopy, the BCPi was assessed again, and whether the operative lung collapsed properly in the operative field was evaluated by the surgeon according to the scale described in the study by Li et al. [13]: 1 = Extremely poor, no lung collapse; 2 = Poor, partial lung collapse with interference with surgical exposure; 3 = Good, total lung collapse but with the presence of some residual air; 4 = Excellent, complete lung collapse with perfect surgical exposure. If the degree of lung collapse was scored as 1 or 2 (i.e., the surgeon could not proceed with the surgery), the bronchial cuff was inflated with air, thereby maintaining a BCP of 20–30 cmH_2_O, which was sustained throughout the surgery.

The baseline characteristics and operative details of the study population were collected perioperatively, including age, sex, height, weight, BMI, ASA physical status, diameter of the LMB, operative side (right or left), angle at which the operating table was tilted, anesthesia time, operation time, and average body temperature during the study period (from anesthesia induction to measurement of the last BCP value).

### 2.5. Study Endpoints

The primary outcome was the change in the BCPi when changing the posture from supine to lateral decubitus; that is, the difference in BCP between the supine and lateral positions at the time of BCVi injection. The secondary outcomes were the changes in the PIP and airway compliance (Cdyn = TVe/[PIP-PEEP]) after lateral positioning, the changes in BCPi following video-assisted thoracoscopic surgery (VATS) initiation, the incidence of overinflation with BCPi exceeding 30 cmH_2_O following lateral positioning or VATS initiation, the incidence of patients whose BCVmin changed when changing posture from supine to lateral decubitus (e.g., increase or decrease of the BCVmin following positional change), and the relationship between the BCVi and other variables, including BCPi, airway pressure, airway compliance, and the gap between the patient’s LMB internal diameter and the DLT’s outer diameter.

### 2.6. Sample Size Calculation

In the pilot study, the mean ± standard deviation (SD) of the change in the BCPi was 0.2 ± 0.45 cmH_2_O in the BCVi = 0 group (*n* = 4) and 2.2 ± 3.29 cmH_2_O in the BCVi > 0 group (*n* = 6). Assuming that the two groups did not show a normal distribution but Tukey’s lambda distribution, the g and h values of the BCVi = 0 group were set to 0.7 and 0.1 and those of the BCVi > 0 group were set to 0.1 and 0.1, respectively. This was because many values of 0 were expected in the BCVi = 0 group; moreover, it was assumed that the skewness towards the right side was severe. Using 2000 samples obtained through Monte Carlo sampling, the sample size required to achieve a statistical power of 90% with an alpha error of 5% was obtained as 25 in the BCVi = 0 group and 38 in the BCVi > 0 group, thereby resulting in a total of 63 patients. Considering a dropout rate of 10%, the number of participants required in this study was 70. The sample size was calculated using PASS 15 (NCSS, Kaysville, UT, USA).

### 2.7. Statistical Analysis

The data were analyzed on an intention-to-treat basis, and missing data were handled using the last observation carried forward method. Normally and non-normally distributed data are presented as the mean (SD) or median (interquartile range), respectively, according to the results of the Kolmogorov–Smirnov test. Categorical data are presented as the number of patients (percentage).

Student’s *t*-test or the Mann–Whitney U-test was used to compare variables between the two groups. Categorical data were compared using the χ^2^ test or Fisher’s exact test. Pearson’s or Spearman’s correlations were calculated to determine the relationships between changes in the BCVi and other variables. Statistical significance was set at *p* < 0.05. All statistical analyses were performed using SPSS Statistics version 19.0.0 (IBM, Armonk, NY, USA).

## 3. Results

Seventy patients, including 40 in the BCVi > 0 group and 30 in the BCVi = 0 group, were included in the study. Patient and surgical characteristics are presented in Table 1 and Table 2. One patient in the BCVi > 0 group and three patients in the BCVi = 0 group showed an increase in BCVmin after lateral positioning (Table 3), indicating that air leak around the bronchial cuff occurred when air from the BCVi was injected in the lateral position. Since the BCP at the time of the air leak was not measured, these patients could not be included in the analysis of the changes in the BCPi. Thus, the analysis of the main outcome of the study, including BCPi, PIP, and Cdyn, was conducted using data from the rest of the patients: BCVi > 0 group, 39; and BCVi = 0 group, 27. (Table 3). 

Considering the primary outcome, the changes in the BCPi after lateral positioning (i.e., ∆BCPi from supine to lateral position) were significantly larger in the BCVi > 0 group than in the BCVi = 0 group (1.5 (0.5–6.0) cmH_2_O vs. 0.0 (0.0–1.0) cmH_2_O; *p* < 0.001) (Table 4). The changes after starting VATS (i.e., ∆BCPi from lateral position to the start of the VATS) were also significantly larger in the BCVi > 0 group than the BCVi = 0 group (3.5 ± 3.4 cmH_2_O vs. 2.0 ± 1.7 cmH_2_O; *p* = 0.036) (Table 4). The BCPi values at each study timepoint (supine position, lateral position, and VATS initiation) were higher in the BCVi > 0 group than in the BCVi = 0 group (all *p* < 0.001) (Table 4).

The incidences of overinflation exceeding 30 cmH_2_O were 5.1% and 10.3% following lateral positioning and VATS initiation, respectively, in the BCVi > 0 group; no patients with overinflation were observed in the BCVi = 0 group. However, no significant difference was observed between the two groups (Table 4).

Considering the variables related to BCVi, a significant correlation was observed with the LMB diameter (Spearman’s rho = 0.676, *p* < 0.001) and the gap between the LMB diameter and the outer diameter of the bronchial cuff (Spearman’s rho = 0.553, *p <* 0.001).

## 4. Discussion

The present study has demonstrated that the change in the BCP of the DLT owing to a positional change from the supine to the lateral decubitus position could be affected by the BCVi, that is, whether the bronchial cuff was inflated at the initial state in the supine position. Upon dividing the patients into two groups based on whether the BCVi was 0 or greater than 0, the change in the BCPi after lateral positioning was significantly smaller in the BCVi = 0 group than in the BCVi > 0 group. In addition, the change in the BCPi following VATS initiation was significantly smaller in the BCVi = 0 group than in the BCVi > 0 group.

The influence of gravity-induced morphological and conformational changes of the trachea on the change in cuff pressure of the endotracheal tube by position (e.g., head-down tilt) has already been explained in previous studies [14]. In addition, in our previous study, we explained that anatomical changes related to the curvature and length of the LMB, as well as the gravitational effect of surrounding structures, could be factors involved in the mechanism of the effect of lateral positioning on the BCP change of the DLT [2]. According to Boyle’s law, the pressure exerted by a given gas mass is inversely proportional to the volume occupied by a closed system when the temperature is constant [15]. Under this principle, compression and stretching of the LMB by surrounding structures caused by the postural change or the VATS procedure reduce the volume occupied by the bronchial cuff within the LMB, thereby increasing the BCP. However, when the bronchial cuff is not inflated, the effect on the change in the cuff pressure appears to be minimal because the bronchial cuff has room to maintain its original volume even if the space within the LMB is reduced.

Since the bronchial cuff plays an important role in lung isolation during OLV, maintaining a pressure above the lower limit of the optimal range of cuff pressure is not necessary if there is no air leak. Several methods for detecting air leaks exist, such as the positive pressure technique (PPT), negative pressure technique (NPT), and CO_2_ analysis technique (CAT) [4,6,7]. Thus, the methods for obtaining the BCVmin in which air leak does not occur may be diversified. Considering the methods introduced in previous literature, the airway pressure artificially applied to detect leaks varies based on the study; however, it is generally established at 25–30 cmH_2_O as a standard range. Recent studies have used CO_2_ analysis (i.e., the capnogram waveform-guided method) and have demonstrated that the minimum cuff volume without air leak obtained using this method is lower than the volume obtained according to the recommended range of cuff pressure [6,7]. Unlike the methods of previous studies that detected air leaks with the lumen on the non-ventilated lung side, in the present study, air leak was confirmed using a capnogram and a spirometer on the ventilated lung side. The method using a spirometer is reportedly a more accurate method that can monitor in real-time and detect even a small amount of air leak [16,17]. In this study, 30 patients were recruited to the BCVi = 0 group using this method, in which an airtight seal can be obtained without inflating the bronchial cuff. Compared to the BCVi = 0 group, changes in the BCPi seen in the BCVi > 0 group, in which the bronchial cuff is inflated, can be considered clinically insignificant. However, during lateral positioning or VATS initiation, which is essential for surgery, the cuff pressure initially set as the optimal range may increase; thus, unintentionally exceeding the upper limit of the optimal range of the cuff is possible. Considering the possibility of overinflation, the incidence of cuff pressure exceeding 30 cmH_2_O was different between the two groups; however, it was statistically insignificant and no such cases were observed in the BCVi = 0 group. Considering these results, it may be useful not to inflate the bronchial cuff unless there is an air leak during OLV.

BCVi, the volume of the bronchial cuff that must be inflated to prevent air leakage, was shown to be related to the gap between the patient’s LMB diameter and the outer diameter of the bronchial cuff in the present study. Therefore, to minimize changes in the BCPi, due to changes in posture or procedure, establishing a standardized method for selecting a DLT size with a bronchial cuff that fits each patient’s LMB is helpful as it allows the BCVi to be minimal. Several previous studies have also recommended that the largest DLT that can atraumatically enter the bronchus should be selected when selecting the proper DLT size [18,19]. The LMB diameter can be estimated or measured using radiologic imaging, such as chest radiography or CT, for selecting an appropriately sized DLT [20,21]. In particular, Hannallah et al. demonstrated that LMB measurement using CT can be a useful method for selecting DLT size [21]. However, in previous studies, including Hannallah et al.’s, when no air leak was observed despite the complete deflation of the bronchial cuff, it was considered an oversized tube as it was presumed to be tightly wedged in the bronchus [4,10,21,22]. These studies differ from our study in that PPT with artificially applied 25–30 cmH_2_O was used to detect air leaks [4,10,21,22]. Our patients’ average peak airway pressure did not exceed 25 cmH_2_O in either group, which is different from the condition judged to be using an oversized tube following the existing guidelines. Despite the need for a standardized DLT size selection method, as mentioned above, implementing it has inherent difficulties. In previous reports, the LMB diameter was measured, and a method of selecting a size of 1 to 2 mm smaller or with an allowable difference of 1.1 to 1.6 mm was used, but the cases where an inappropriately sized DLT was selected could not be avoided entirely [10,21]. This is because the LMB diameter measurement on CT may differ from the actual measurement, and the external diameter may be inconsistent even for DLTs of the same size [10]. Therefore, in most cases where these measurement errors make it impossible to select a DLT for LMB and an increase in BCVi is unavoidable, it is necessary to routinely monitor BCP during thoracic surgery, recognizing that BCP may increase during lateral positioning and VATS initiation.

This study had certain limitations. First, the uniform application of the results of this study to a wide spectrum of patients is challenging because only participants with normal lung function were included. For example, the airway pressure during OLV can be excessively high based on lung function; under these conditions, it would be difficult to find patients in the BCVi = 0 group, that is, patients in whom air leakage does not occur without inflating the bronchial cuff. If they were enrolled in the BCVi = 0 group at such a high pressure, it is assumed that the patient was given an oversized tube, tightly wedged in the bronchus. Second, since the sample size was calculated based on the primary endpoint, the number of samples may not be sufficient to analyze other outcomes. Moreover, a subgroup analysis could not be performed for the direction of the lateral position (left or right). However, as no significant difference was observed in the number of patients corresponding to each positional direction between the two groups, the bias caused by the direction is expected to be minimal. Finally, since the observational period in our study was until the start of VATS and the subsequent periods were not included in the study, outcomes of the subsequent intraoperative and postoperative periods were omitted from the study. In prolonged surgery, in which considerable manipulation of the lungs and bronchi occurs, the resulting airway edema and hypersecretion can affect cuff pressure and related ventilatory variables. At our institution, the duration of VATS varies widely based on the type of surgery; thus, these variables can be affected. Therefore, further studies that control the type and time of surgery to some extent are needed for postoperative outcomes, such as the relationship between BCP and postoperative sore throat, which we have not addressed thus far.

## 5. Conclusions

In conclusion, whether the bronchial cuff was initially inflated for a state in which air leaks did not occur could affect the degree of BCP change during lateral positioning; when the BCVi was 0, the degree of change in BCP was significantly smaller than when it exceeded 0. Even if the results of such changes in BCP appear to be clinically insignificant, the risk of unintentionally exceeding the upper limit of the cuff’s optimal range is not completely eliminated. Therefore, considering BCVi was related to the gap between the patient’s LMB diameter and the outer diameter of the bronchial cuff, selecting a DLT size with a bronchial cuff that fits each patient’s LMB is thought to be useful in minimizing the change in BCP when performing lateral positioning during thoracic surgery. In addition, it is necessary to be aware that BCP may increase at the time of performing lateral positioning or starting VATS, if the bronchial cuff needs to be initially inflated; thus, consideration should be given to monitoring BCP routinely during thoracic surgery, especially during lateral positioning and VATS initiation, if possible.

## Figures and Tables

**Table 1:** Baseline patient characteristics.

	BCVi > 0 Group	BCVi = 0 Group	*p*-Value
	(*n* = 39)	(*n* = 27)	
Age (years)	59 (18–80)	54 (18–79)	0.505
Sex (male/female)	32/7	15/12	0.028
Weight (kg)	64.5 ± 8.9	60.2 ± 10.5	0.076
Height (cm)	169.2 ± 9.0	164.3 ± 11.1	0.055
BMI (kg/m^2^)	22.7 ± 3.4	22.3 ± 3.4	0.695
ASA physical status (1/2/3)	10/28/1	6/18/3	0.474
Side of surgery (right/left)	24/15	16/11	1.000
FEV1/FVC (%)	75.5 ± 7.3	76.2 ± 6.4	0.743
FVC of the predicted (%)	96.8 ± 12.0	105.1 ± 17.0	0.064
Diameter of the LMB (mm)	12.1 ± 0.7	10.7 ± 0.9	0.000

Data are presented as the median (range), number, or mean ± standard deviation. BCVi, initially established minimum bronchial cuff volume without air leak measured in the supine position; BMI, body mass index; ASA, American Society of Anesthesiologists; FEV1, forced expiratory volume in one second; FVC, forced vital capacity; LMB, left main bronchus.

**Table 2:** Operative details.

	BCVi > 0 Group	BCVi = 0 Group	*p*-Value
	(*n* = 39)	(*n* = 27)	
Anesthesia time (min)	120.0 (81.0–161.0)	89.0 (63.0–157.0)	0.259
Operation time (min)	86.0 (48.0–137.0)	59.0 (30.0–125.0)	0.190
Body temperature (°C)	36.5 ± 0.3	36.5 ± 0.2	0.321
Table inclination angle of head side (degree)	11.8 ± 5.7	12.8 ± 5.9	0.489
Type of surgery (lobectomy/wedge resection/mediastinal surgery)	17/19/3	11/15/1	1.000
Degree of lung collapse (1/2/3/4)	0/2/29/8	0/3/21/3	0.304

Data are presented as the median (interquartile range), mean ± standard deviation, or number. BCVi, initially established minimum bronchial cuff volume without air leak measured in the supine position.

**Table 3:** Outcomes related to bronchial cuff volume.

	BCVi > 0 Group	BCVi = 0 Group	*p*-Value
	(*n* = 40)	(*n* = 30)	
BCVi (mL)			
0		30 (100)	
0.5	29 (72.5)		
1.0	10 (25.0)		
1.5	1 (2.5)		
Change in BCVmin during lateral positioning			0.457
No change	29 (72.5)	27 (90.0)	
Decrease	10 (25.0)	0 (0.0)	
Increase	1 (2.5)	3 (10.0)	

Data are presented as a number (percentage). BCVmin, the smallest bronchial cuff volume without air leak; BCVi, initial established BCVmin measured in the supine position.

**Table 4:** Outcomes of bronchial cuff pressure and ventilatory variables.

	BCVi > 0 Group	BCVi = 0 Group	*p*-Value
	(*n* = 39)	(*n* = 27)	
BCPi (cmH_2_O)			
∆ (from supine to lateral)	1.5 (0.5–6.0)	0.0 (0.0–1.0)	<0.001
∆ (from lateral to VATS)	3.5 ± 3.4	2.0 ± 1.7	0.036
Supine position	15.9 ± 6.7	0.3 ± 0.6	<0.001
Lateral position	18.8 ± 6.7	0.6 ± 0.8	<0.001
VATS operation	22.4 ± 7.1	2.7 ± 1.9	<0.001
Overinflation; BCPi > 30 cmH_2_O			
After lateral positioning	2 (5.1)	0	0.509
After VATS starting	4 (10.3)	0	0.138
PIP (cmH_2_O)			
∆ (from supine to lateral)	0.0 (−1.0–2.0)	0.0 (0.0–1.0)	0.791
Supine position	20.8 ± 2.7	21.3 ± 3.5	0.565
Lateral position	21.6 ± 3.5	21.9 ± 3.5	0.733
Cdyn (mL/cmH_2_O)			
∆ (from supine to lateral)	0.0 (−1.9–3.0)	−0.6 (−2.1–1.7)	0.348
Supine position	22.5 ± 4.0	23.3 ± 6.7	0.559
Lateral position	22.9 ± 4.8	22.2 ± 4.5	0.564

Data are presented as the median (interquartile range), mean ± standard deviation, or number (percentage). BCVi, initially established minimum bronchial cuff volume without air leak measured in the supine position; BCPi, bronchial cuff pressure when injecting the BCVi; VATS, video-assisted thoracoscopic surgery; PIP, peak inspiratory pressure; Cdyn, airway compliance.

## Data Availability

The data presented in this study are available on request from the corresponding author.
